# *NBS1* rs2735383 polymorphism is associated with an increased risk of laryngeal carcinoma

**DOI:** 10.1186/s12885-018-4078-2

**Published:** 2018-02-12

**Authors:** Xinmei Hu, Juan Liao, Huiliu Zhao, Feng Chen, Xuefeng Zhu, Jiangheng Li, Qingqing Nong

**Affiliations:** 1 School of Public Health, Guangxi Medical University, 22 Shuangyong Road, Nanning, Guangxi 530021 China; 2 Department of Clinical Laboratory, The Affiliated Tumor Hospital of Guangxi Medical University, Nanning, Guangxi China; 3 Department of Medical Oncology Division, The First Affiliated Hospital of Guangxi Medical University, Nanning, Guangxi China; 4 Guangxi Colleges and Universities Key Laboratory of Prevention and Control of Highly Prevalent Diseases, Guangxi Medical University, Nanning, 530021 China

**Keywords:** *NBS1*, Laryngeal carcinoma, Polymorphism

## Abstract

**Background:**

Nijmegen breakage syndrome 1 (*NBS1*), as a key protein in the DNA double-strand breaks (DSBs) repair pathway, plays an important role in maintaining genomic stability. Although single nucleotide polymorphisms (SNPs) in *NBS1* have frequently been studied in multiple cancers, the relationships of two functional *NBS1* polymorphisms (rs2735383 and rs1805794) with laryngeal carcinoma are yet unclear. Therefore, in the present study, we performed a case-control study including 342 cases and 345 controls to analyze the associations between two polymorphisms of *NBS1* and the risk of laryngeal carcinoma.

**Methods:**

We used the polymerase chain reaction-restriction fragment length polymorphism (PCR-RFLP) method to determine the genotypes of the functional SNPs in *NBS1* gene.

**Results:**

In comparison with the homozygous rs2735383GG genotype, the CC genotype was significantly associated with an increased risk of laryngeal carcinoma (adjusted OR = 1.884, 95%CI = 1.215–2.921). The rs2735383C variant genotypes (GC + CC) conferred a 1.410-fold increased risk of laryngeal carcinoma (adjusted OR = 1.410, 95%CI = 1.004–1.980). Furthermore, when compared to rs2735383GG genotype in laryngeal carcinoma tissues, the combined GC and CC genotypes exerted a significantly lower mRNA level of *NBS1* (*P* = 0.003). In contrast, no significant association was found between rs1805794G > C polymorphism and cancer risk (adjusted OR = 1.074, 95%CI = 0.759–1.518 for GC; adjusted OR = 1.100, 95%CI = 0.678–1.787 for CC; adjusted OR = 1.079, 95%CI = 0.774–1.505 for GC + CC).

**Conclusions:**

These findings indicate that rs2735383G > C polymorphism in *NBS1* may play a crucial role in the development of laryngeal carcinoma.

**Electronic supplementary material:**

The online version of this article (10.1186/s12885-018-4078-2) contains supplementary material, which is available to authorized users.

## Background

Laryngeal carcinoma is one of the most common malignant tumors. According to the data from International Agency for Research on Cancer, it accounts for 30% to 40% of head and neck malignancies and 1% to 2.5% of all malignant neoplasms in human, respectively [1]. Although the overall incidence is declining [2], laryngeal cancer is still an unignorable health issue throughout the world. In the Netherlands, about 700 people are diagnosed with laryngeal carcinoma annually [3]. In China, the incidence of laryngeal carcinoma is only secondary to nasopharyngeal carcinoma among all types of head and neck cancers. In some regions, its incidence is even the highest [4].

Epidemiological studies have established many etiologic factors of laryngeal cancer, including smoking and alcohol consumption [5]. These above factors can potentially modify DNA, cause genomic instability, and thus lead to carcinogenesis. Efficient and accurate repairs of DNA damage could maintain the stability of genome. However, deficiencies of DNA double-strand breaks (DSBs) repair might sensitize carcinogens induced genomic instability and cancer [6]. The repair of DSBs in human cells includes two different pathways: homologous recombination (HR) and non-homologous end-joining (NHEJ) pathways [7–9]. As a component of MRE11-RAD50-NBS1 (MRN) complex, Nijmegen breakage syndrome 1 (*NBS1*) plays a key role in the DSBs repair pathway and participates in both HR pathway and NHEJ pathway [10]. Several reports have described the associations between SNPs in *NBS1* and human cancer risks [11–14]. However, the association of *NBS1* gene’s variations with laryngeal carcinoma has been rarely substantiated. Ziólkowska I et al. demonstrated that heterozygous carriers of c.I171V variant were prone to the development of larynx cancer [15]. Nowak J et al. reported that *NBS1* g.657del5 contributed significantly to a higher risk of laryngeal carcinoma [16]. However, the frequencies of these loci are rare in Chinese. Nevertheless, these data suggested the *NBS1* might be a susceptible gene of laryngeal carcinoma.

As one of the most commonly studied polymorphisms in *NBS1*, rs1805794 (c.553G > C) has been shown to increase susceptibility in multiple cancers, such as acute myeloid leukemia [17], hepatic cancer [18] and lung cancer [19]. The transition of G to C resulted in reduced DNA repair capacity of *NBS1* and promoted tumor migration [19, 20]. Another well-studied SNP rs2735383 (g.90947269G > C) in the 3’-UTR of *NBS1* has also been studied for multiple times [13]. Recent studies found that rs2735383 was associated with substantially increased risk of colorectal cancer [21] and lung cancer [22]. This SNP was also functional by decreasing *NBS1* expression [22]. However, the functions of these two variants on laryngeal carcinoma were yet unclear. Thus, we hypothesized that rs1805794G > C and rs2735383G > C might affect the DNA repair ability of *NBS1* and contribute to laryngeal carcinoma.

In this study, we performed a case-control study to investigate the association between these two polymorphisms of *NBS1* and the risk of laryngeal carcinoma in Han or Zhuang population in Guangxi Province of China.

## Methods

### Study subjects

In this study, 342 patients with histopathologically diagnosed primary laryngeal carcinoma were recruited between 2014 and 2016 in the Affiliated Tumor Hospital of Guangxi Medical University. They had a response rate of 95% among all cancer patients diagnosed in the hospital. Clinical and pathological information on all laryngeal carcinoma diagnoses were confirmed by manual review of the pathology reports and endoscopic findings of Otorhinolaryngology Department. Of the 342 cases, 37 were poorly differentiated squamous cell carcinoma (SCC), 63 were moderately differentiated SCC, 76 were well-differentiated SCC, and 166 remain unknown. Totally, 345 cancer-free controls who have matched age (±5 years) and sex with the cases were recruited from the same hospital. These control individuals had a response rate of 84%. We only recruited people whose ethnicity is Han or Zhuang.

After having given a written informed consent, all individuals were interviewed according to a structured questionnaire in order to collect personal information including age, sex, smoking status, drinking status and so on. The participants who have smoked less than 100 cigarettes in their lifetime were defined as never-smokers; otherwise were defined as ever-smokers [23]. Similarly, the participants who have consumed alcohol at least once a week for more than one year were defined as alcohol ever-drinkers and the remaining as alcohol never-drinkers [24]. Each study participant was asked to donate a one-time blood sample of 5 ml for later examination. This study was approved by the Medical Ethics Committee of Guangxi Medical University (GXMU-20140307-4).

### Genotyping analysis

Based on the previous studies [25–27], we selected and genotyped two *NBS1* SNPs (rs1805794G > C in exon 5 and rs2735383G > C in 3’-UTR). According to the dbSNP database, the current study defined the C allele of both SNPs in the antisense strand (i.e., the corresponding allele is G in the sense strand in the database) as minor allele [20, 22]. We used the polymerase chain reaction-restriction fragment length polymorphism (PCR-RFLP) method in Zheng’s study [20] to verity the association between *NBS1* polymorphism and laryngeal carcinoma risk.

The primer pairs used to amplify the DNA fragment containing the rs1805794G > C polymorphism were 5’-ACCTT TCAAT TTGTG GAGGC-3′ (forward) and 5’-AGTCG GTCTT TGGTC ACTGC-3′ (reverse), to produce a fragment of 289 bp. The primer pairs for rs2735383G > C were 5’-TGCAG TGTTC TACAC CTTGC TT-3′ (forward) and 5′-AGGTG ACATC TGCAC CACTG-3′ (reverse), to produce a fragment of 156 bp. PCR was performed in a 25 μl reaction system, containing 5 mM MgCl_2,_ 0.1 mM deoxynucleotide triphosphates, 3.0 U of Taq polymerase (MBI Fermentas, Vilnus, Lithuania), and the manufacturer’s buffer. After an initial melting step at 94 °C for 5 min, the PCR procedure consists of 35 cycles including denature at 94 °C for 45 s, anneal at 62.9 °C for rs1805794 or 61.0 °C for rs2375383 for 45 s, and extension at 72 °C for 45 s, followed by a final extension step at 72 °C for 7 min. The amplified fragment containing rs1805794 polymorphism was cut by *Hin*f1 (Takara BioTech, Dalian, China) at 37 °C for at least 3 h. The major G allele produced a single 289 bp band, while the minor C allele produced 30- and 259-bp bands. The digestion products were separated and visualized in 3% agarose gel electrophoresis. Meanwhile, the amplified fragment containing rs2735383 polymorphism was cut by *Cvi*QI (New England Biolabs, Beijing, China) at 25 °C for at least 3 h. The minor G allele produced a single 156-bp band, while the major C allele produced two separate bands of 57-bp and 99-bp.

### Detection of *NBS1* mRNA expression by real-time PCR

Total RNA from 32 laryngeal carcinoma tissues were extracted using TriPure Reagent (Roche Applied Science) and reverse transcribed to complementary DNA using cDNA synthesis kit ThermoScrept™ RT-PCR System (Invitrogen). The relative mRNA expression levels of *NBS1* covering rs2735383 site and *β*-actin (as an internal reference) were measured by the ABI Prism 7500 Sequence Detection Systems (Applied Biosystems) using the SYBR-Green method, with the same baseline and threshold set for each plate to generate threshold cycle (Ct) values for both genes in each sample. The primers used for detection of *NBS1* were as follows: 5′-TTGGT TGCAT GCTCT TCTTG-3′ (forward) and 5’-GGCTG CTTCT TGGAC TCAAC-3′ (reverse); and for *β*-actin 5’-GGCGG CACCA CCATG TACCC T-3′ (forward) and 5′-AGGGG CCGGA CTCGT CATAC T-3′ (reverse). The 2 ^-△△Ct^ method was used to measure the level of *NBS1* gene’s expression.

### Statistical analysis

We used Chi-square (*χ*^2^) test to analyze the differences of selected characteristics (age, sex, ethnicity, smoking status and drinking status) between cases and controls. Hardy-Weinberg equilibrium (HWE) was tested using a goodness-of-fit *χ*^2^ test by comparing the expected genotype frequencies with that of the controls. The associations between the two polymorphisms of *NBS1* and risk of laryngeal carcinoma were respectively calculated by odds ratios (ORs) with 95% confidence intervals (CIs) using a unconditional logistic regression model, after adjusting age, sex, smoking status and drinking status. Student’s *t*-test was used to evaluate the differences in *NBS1* mRNA expression between different groups. All tests were analyzed using the Stata 13.0 software (Stata Corp LP, College Station, Texas, United States). The statistical power was calculated by using the PS Software (http://biostat.mc.vanderbilt.edu/twiki/bin/view/Main/PowerSampleSize). *P* < 0.05 was considered statistically significant.

## Results

### Associations between the *NBS1* SNPs and laryngeal carcinoma risk

In order to determine whether the rs1805794 and rs2735383 polymorphisms of *NBS1* gene are associated with laryngeal carcinoma, we examined the genotypic distribution of these two polymorphisms of *NBS1* in both cases and controls. In this study, 342 laryngeal carcinoma cases and 345 controls were examined. The basic characteristics of laryngeal carcinoma cases and controls were shown in Table 1. There were no significant differences in distributions of sex, age, and ethnicity between cases and controls (*P* > 0.05 for all), while the smoking status and drinking status were significantly different between cases and controls (*P* = 0.024 and *P* = 0.000 respectively).

**Table 1:** The selected characteristics between laryngeal carcinoma cases and controls used for association study

	Cases(*n* = 342)	Controls(*n* = 345)	*P* ^a^
	N (%)	N (%)	
Sex			
Male	333(97.368)	334(96.812)	0.664
Female	9(2.632)	11(3.188)	
Age(years)			
<30	1(0.292)	2(0.580)	0.864
30–59	150(43.860)	154(44.638)	
60–89	191(55.848)	188(54.492)	
≥ 90	0(0.000)	1(0.290)	
Ethnicity			
Han	231(67.544)	238(68.986)	0.685
Zhuang	111(32.456)	107(31.014)	
Smoking status			
ever	223(65.205)	196(56.812)	0.024
never	119(34.795)	149(43.188)	
Drinking status			
ever	176(51.462)	100(28.986)	0.000
never	166(48.538)	245(71.014)	
Classification of diagnosis			
poorly differentiated squamous cell carcinoma	37(10.819)		
Moderately differentiated squamous cell carcinoma	63(18.421)		
well-differentiated squamous cell carcinoma	76(22.222)		
Unknown	166(48.538)		

^a^A χ^2^ test for differences in selected characteristics between cases and controls

The genotype distributions of rs1805794 and rs2735383, and their respective associations with laryngeal carcinoma risk are summarized in Table 2. The observed genotype frequencies of these two SNPs among the control subjects were all in agreement with the Hardy-Weinberg Equilibrium (*P* = 0.081 for rs1805794 and *P* = 0.963 for rs2735383). As shown in Table 2, the respective frequencies of rs2735383 GG, GC and CC genotypes in the cases were 26.608%, 47.076%, 26.316%, differing significantly from 33.043%, 48.986%, 17.971% in the controls (*P* = 0.019). The logistical model showed that rs2735383CC variant genotype conferred a significantly increased risk of laryngeal carcinoma in comparison with the common genotype of GG (adjusted OR = 1.884, 95% CI =1.215–2.921), but the GC genotype fails (adjusted OR = 1.236, 95% CI =0.861–1.775). When combining these two genotypes, the rs2735383C variant genotypes (GC + CC) contributed to a 1.410-fold increased risk of laryngeal carcinoma in comparison to that of the GG genotype (adjusted OR = 1.410, 95% CI =1.004–1.980). The C allele of rs2735383 in a dose-dependent model was associated with an increased cancer risk (*P*_trend_ = 0.005).

**Table 2:** Frequency distribution of genotypes in *NBS1* and results of logistic regression analysis for their associations with laryngeal carcinoma risk

Genotypes	cases	controls^a^	*P* ^b^	Crude OR (95%CI)	Adjusted OR (95%CI)^C^
	N(%)	N(%)			
Total no. of subjects	342	345			
rs1805794(G > C)					
GG	105 (30.702)	111 (32.174)	0.917	1.000 (ref)	1.000 (ref)
GC	185 (54.094)	183 (53.043)		1.069 (0.764–1.496)	1.074 (0.759–1.518)
CC	52 (15.205)	51 (14.783)		1.078 (0.674–1.724)	1.100 (0.678–1.787)
Trend test *P* value				0.707	0.660
GC + CC	237 (69.298)	234 (67.826)		1.071 (0.776–1.478)	1.079 (0.774–1.505)
rs2735383(G > C)					
GG	91 (26.608)	114 (33.043)	0.019	1.000 (ref)	1.000 (ref)
GC	161 (47.076)	169 (48.986)		1.193 (0.841–1.694)	1.236(0.861–1.775)
CC	90 (26.316)	62 (17.971)		1.819 (1.189–2.781)	1.884 (1.215–2.921)
Trend test *P* value				0.007	0.005
GC + CC	251 (73.392)	231 (66.957)		1.361 (0.980–1.890)	1.410(1.004–1.980)

^a^The observed genotype frequencies of the two SNPs among the control subjects were all in agreement with the Hardy-Weinberg equilibrium(*P* > 0.05 for all)

^b^A χ^2^ test for differences in distribution of genotype frequencies between cases and controls

^c^Adjusted in a unconditional logistic regression model that included age, sex, smoking status and drinking status

In contrast, no significant association was observable between rs1805794G > C polymorphism and the risk of laryngeal carcinoma (*P* = 0.917, adjusted OR = 1.074, 95% CI = 0.759–1.518 for GC; adjusted OR = 1.100, 95% CI = 0.678–1.787 for CC; adjusted OR = 1.079, 95% CI = 0.774–1.505 for GC + CC). Combined analyses were conducted to evaluate the cumulative effect of rs1805794 and rs2735383 (Additional file 1). In comparison with the homozygous genotypes, the combined variant genotypes were not significantly associated with increased laryngeal carcinoma risk. Thus, our study demonstrated that *NBS1* SNPs rs2735383G > C might be associated with the elevated risk of laryngeal carcinoma. We then focused on SNP rs2735383 in the following analyses.

### Stratification analysis

Next, we tried to stratify the subjects according to different factors and then determined the effects of SNP rs2735383 on laryngeal carcinoma risk. As showed in Table 3, the rs2735383C variant genotypes (GC + CC) were significantly associated with an increased risk of laryngeal carcinoma in the male subgroup (adjusted OR = 1.505, 95%CI =1.066–2.126) and the ever-smoker subgroup (adjusted OR = 1.602, 95%CI = 1.023–2.510). But we failed to observe any significant interactions between *NBS1* polymorphisms and these two confounding factors (all *P*_interaction_ > 0.05), possibly due to the limited sample size in each subgroup.

**Table 3:** Stratification analysis of the *NBS1* rs2735383 genotypes by selected variables in laryngeal carcinoma cases and controls

	cases(n = 342)		controls (n = 345)		Adjusted OR (95%CI)^a^	*P*_interaction_^b^
	GG	GC + CC	GG	GC + CC	GC + CC vs GG	
	N (%)	N (%)	N (%)	N (%)		
Age						
≤ 60	49 (14.327)	113 (33.041)	61 (17.681)	109 (31.594)	1.306 (0.811–2.106)	0.080
>60	42 (12.281)	138 (40.351)	53 (15.362)	122 (35.363)	1.496 (0.919–2.436)	
Sex						
Male	87 (25.439)	246 (71.930)	113 (32.754)	221 (64.058)	1.505 (1.066–2.126)	0.762
Female	4 (1.170)	5 (1.461)	1 (0.290)	10 (2.898)	0.127 (0.010–1.549)	
Ethnicity						
Han	62(18.129)	169(49.415)	81(23.478)	157(45.507)	1.439(0.954–2.169)	0.526
Zhuang	29(8.480)	82(23.977)	33(9.565)	74(21.449)	1.394(0.755–2.573)	
Smoking status						
Ever	62 (18.129)	161 (47.076)	73 (21.159)	123 (35.652)	1.602 (1.023–2.510)	0.152
Never	29 (8.480)	90 (26.316)	41 (11.884)	108 (31.304)	1.191 (0.681–2.083)	
Drinking status						
Ever	53 (15.497)	123 (35.965)	33 (9.565)	67 (19.420)	1.225 (0.703–2.133)	0.907
Never	38 (11.111)	128 (37.427)	81 (23.478)	164 (47.536)	1.594 (0.985–2.514)	

^a^ORs were adjusted for age, sex, smoking status, drinking status

^b^*P* value of a test the multiplicative interaction between rs2735383 and selected variables on cancer risk were calculated using logistic regression models

### Association of the rs2735383G > C genotype and *NBS1* mRNA expression level

**Fig. 1:**
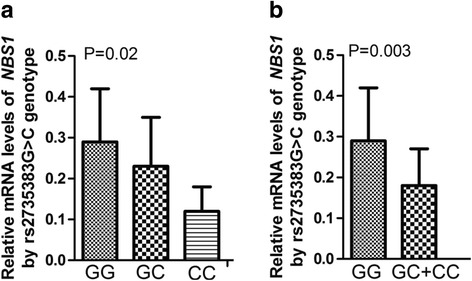
The relative mRNA levels of *NBS1* by rs2735383G > C genotype. **a** The differences in the mRNA expression levels were analyzed by ANOVA test. **b** After combined GC and CC, the differences in the expression levels were analyzed by Student’s *t*-test

As shown in Fig. 1(a), among 32 laryngeal carcinoma tissues examined, the tissues of CC genotype have significantly lower mRNA expression (0.12 ± 0.06 for 7 cases with CC) of *NBS1* than those with GC or GG genotype (0.23 ± 0.12 for 16 cases with GC and 0.29 ± 0.13 for 9 cases with GG; ANOVA test: *P* = 0.02). Furthermore, as seen in Fig. 1(b), the dichotomized analysis showed that the mRNA levels of *NBS1* from those tissues with combined GC and CC genotypes (0.18 ± 0.09 for 23 cases with GC + CC) were significantly lower than that of GG genotype (0.29 ± 0.13 for 9 cases with GG; Student’s *t*-test: *P* = 0.003).

## Discussion

In the present study, we analyzed the associations between two common *NBS1* SNPs (rs1805794G > C and rs2735383G > C) and laryngeal carcinoma risk in 342 laryngeal carcinoma cases and 345 controls. We found that the rs2735383C variant genotypes (GC + CC) contributed an increased risk to laryngeal carcinoma. The risk also increased as the number of rs2735383C allele increased. The *NBS1* mRNA expression level of the rs2735383CC genotype was lower in laryngeal carcinoma tissues compared to that of GC or GG genotype. Thus, our study validated the hypothesis that rs2735383G > C polymorphism of *NBS1* gene was associated with the increased risk of laryngeal carcinoma. In contrast, there was no significant difference for rs1809754G > C variant compared to common homozygous genotype.

*NBS1* is one of the DNA homologous recombination repair protein, and belongs to the MRE11-RAD50-NBS1 complex. It plays a crucial role in maintaining genomic stability because the hMRE11/hRAD50/NBS1 protein complex functions as a sensor to detect DNA damage [10]. Mutations in *NBS1* may cause Nijimegen breakage syndrome (NBS), which is characterized by radiosensitivity, immunodeficiency, chromosomal instability and an increased risk for cancer [28]. Multiple studies have established the essential roles of *NBS1* protein in DSBs repair and tumor development in multiple types of tumor, including ovarian tumors [25], lung cancer [26], non-Hodgkin lymphoma [29] and sporadic breast cancer [30].

*NBS1* rs1805794 polymorphism, which has frequently been studied, is associated with risks of breast cancer [27], cervix carcinoma [31], nasopharyngeal carcinoma [20], and bladder cancer [32]. Moreover, functional studies have demonstrated that the rs1805794G > C is functional and could recede the DNA repair capacity of *NBS1* [19]. This transition also impairs *NBS1*’s capacity of inhibiting tumor invasion [20]. However, *NBS1* rs1805794G > C is not always associated with cancer risk. For example, a study found that the rs1805794 variant genotype was not associated with the risk of some tumors, including head and neck cancers in a Polish population [33]. Similarly, our present results also showed that the polymorphism rs1805794G > C was not associated with the risk of laryngeal carcinoma. Some researchers found that rs1805794GC conferred a 1.68-fold risk of larynx cancer compared with GG genotype, which is different from our study [15]. The reason for such discrepancies in results may be that our study is only related to Han and Zhuang Chinese.

The other examined rs2735383G > C polymorphism locates in the 3’-UTR of *NBS1*. Yang et al. demonstrated that rs2735383G > C was functional by decreasing *NBS1* expression via formation of a novel binding site of microRNA-629 to the 3’-UTR of *NBS1* gene [22]. Some researchers found that rs2735383G > C in *NBS1* was significantly associated with the risks of colorectal cancer [21] and lung cancer [22]. Although some studies revealed an insignificant association between rs2735383G > C and breast cancer risk [34–36], this SNP was significantly associated with progestrone receptor positivity of breast cancer patients [36]. The present study is the first to investigate the association between *NBS1* rs2735383 and the risk of developing laryngeal carcinoma. Our results supported the conclusion that the *NBS1* rs2735383 polymorphism was associated with laryngeal carcinoma risk. In addition, c.I171V and g.657del5 have been reported to be risk loci of laryngeal carcinoma in other ethnics [15, 16], but we did not test these two loci because they are rare in Chinese. All the above results suggest that the role of *NBS1* polymorphism in laryngeal carcinoma risk may be affected by some ethnic difference, which warrants further investigations.

In this study, we investigated the associations between the SNPs (rs2735383 and rs1805794) and the risks of laryngeal carcinoma. We also took into consideration the relevant factors of age, sex, smoking status and drinking status, all of which might be a major cause of laryngeal carcinoma. However, there are still some limitations in our study. For example, the scale of our study subject group is relatively small. And other factors such as family cancer history may interact with genotype, but this information was unavailable in our case-control study. Due to the fact that our study was a hospital-based case-control one and that our study subjects came from only the Chinese Han and Zhuang ethnicities, some selection biases were unavoidable in this study. Moreover, a considerable proportion of patients were lack of clinical stages and cancer differentiation status, thus limiting our analysis on association between *NBS1* variants and clinical features. Nevertheless, our subject selection met the requirement of randomness because the genotype frequencies among controls fitted the Hardy-Weinberg disequilibrium law. We achieved over 95% study power (two-sided test, α = 0.05) to detect an OR of 1.884 for the rs2735383CC and an OR of 1.410 for the rs2735383 GC + CC, compared to that of the rs2735383GG genotype, suggesting that our findings are well worthy.

In addition, we found that the expression of *NBS1* gene in laryngeal carcinoma tissues differed with genotype of rs2735383. This indicated that the rs2735383 G > C polymorphism is a functional locus. Bioinformatics analysis with the 1000 Genomes Project (https://www.ncbi.nlm.nih.gov/variation/tools/1000genomes/) showed that rs2735383 G > C was in complete linkage disequilibrium with rs1063053G > A and rs1063054 A > C. The prediction using SNPinfo Web Server (https://manticore.niehs.nih.gov/) revealed that these two SNPs are both potently functional. The transition from common allele G of rs1063053 to variant allele A may lose binding site of hsa-miR-517b, and rs1063054A > C could result in new binding site of hsa-miR-654-3p but losing binding site of hsa-miR-513a-3p. These findings further indicate that the functional polymorphism rs2735383G > C in the 3’-UTR of *NBS1* gene could predict the risk of laryngeal carcinoma.

## Conclusions

Taken together, the present study observed a significantly higher frequency of the rs2735383 variant of the *NBS1* gene, indicating that this variant may be a genetic susceptibility factor of laryngeal carcinoma. To the best of our knowledge, this study provided the first evidence that rs2735383G > C polymorphism in *NBS1* is associated with the increased risk of developing laryngeal carcinoma. But these findings need to be verified in future case-control studies of larger sample size and better mechanism-oriented study design.

## Additional file

Additional file 1: **Table S1.** Combined analysis of the cumulative effect of rs1805794 and rs2735383 on laryngeal carcinoma risk. (DOC 34 kb)

## Abbreviations

ANOVA: Analysis of Variance
CI: Confidence interval
DSBs: DNA double-strand breaks
HR: Homologous recombination
HWE: Hardy-weinberg equilibrium
MRN: MRE11-RAD50-NBS1
*NBS1*: Nijmegen breakage syndrome 1
NHEJ: Non-homologous end-joining
OR: Odds ratio
PCR-RFLP: Polymerase chain reaction-restriction fragment length polymorphism
SCC: Squamous cell carcinoma
SNPs: Single nucleotide polymorphisms
