# Supplementary material for: NBS1 rs2735383 polymorphism is associated with an increased risk of laryngeal carcinoma
Source: BMC Cancer. 2018 Feb 12;18:175. doi: 10.1186/s12885-018-4078-2 (PMC5810033; doi:10.1186/s12885-018-4078-2)
Supplement: Supplementary file 1 — Table S1. Combined analysis of the cumulative effect of rs1805794 and rs2735383 on laryngeal carcinoma risk. (DOC 34 kb) [file 12885_2018_4078_MOESM1_ESM.doc]

The combined SNPs (rs2735383 and rs1805794) analysis

| Genotypes | cases | controls^a^ | *χ^2^* | *P value* | OR (95%CI) |
| --- | --- | --- | --- | --- | --- |
|  | N(%) | N(%) |  |  |  |
| rs1805794 GG and rs2735383 GG | 55 (21.484) | 75 (27.778) | 2.797 | 0.094 | 1.000 (ref) |
|  |  |  |  |  |  |
| rs1805794 GC/CC and rs2735383 GC/CC | 201 (78.516) | 195 (72.222) |  |  | 1.406 (0.942-2.097) |

^a^The observed genotype frequencies among the control subjects were all in agreement with the Hardy-Weinberg

equilibrium(*P*>0.05 for all).

A χ^2^ test for differences in distribution of genotype frequencies between cases and controls.
